# Doxorubicin-Loaded Gold Nanoarchitectures as a Therapeutic Strategy against Diffuse Intrinsic Pontine Glioma

**DOI:** 10.3390/cancers13061278

**Published:** 2021-03-13

**Authors:** Caitlin Ung, Maria Tsoli, Jie Liu, Domenico Cassano, Salvador Pocoví-Martínez, Dannielle H. Upton, Anahid Ehteda, Friederike M. Mansfeld, Timothy W. Failes, Annafranca Farfalla, Christopher Katsinas, Maria Kavallaris, Greg M. Arndt, Orazio Vittorio, Giuseppe Cirillo, Valerio Voliani, David S. Ziegler

**Affiliations:** 1 Children’s Cancer Institute, Lowy Cancer Research Centre, University of New South Wales, High Street, Randwick, NSW 2052, Australia; cung@ccia.org.au (C.U.); jliu@ccia.org.au (J.L.); dupton@ccia.org.au (D.H.U.); AEhteda@ccia.org.au (A.E.); fmansfeld@ccia.org.au (F.M.M.); ckatsinas@ccia.org.au (C.K.); mkavallaris@ccia.org.au (M.K.); ovittorio@ccia.org.au (O.V.); 2 School of Women’s and Children’s Health, University of New South Wales, Kensington, NSW 2052, Australia; 3 Center for Nanotechnology Innovation, Istituto Italiano di Tecnologia, Piazza San Silvestro 12, 56127 Pisa, Italy; domenico.cassano@sns.it (D.C.); spocovi@aidimme.es (S.P.-M.); valerio.voliani@iit.it (V.V.); 4 ARC Centre of Excellence in Convergent Bio-Nano Science and Technology, Australian Centre for NanoMedicine, University of New South Wales, Kensington, NSW 2052, Australia; 5 ARC Centre of Excellence in Convergent Bio-Nano Science and Technology, Monash University, Royal Parade, Parkville, VIC 3052, Australia; 6 ACRF Drug Discovery Centre, Children’s Cancer Institute, Lowy Cancer Research Centre, University of New South Wales, High Street, Randwick, NSW 2052, Australia; tfailes@ccia.org.au (T.W.F.); garndt@ccia.org.au (G.M.A.); 7 Department of Pharmacy Health and Nutritional Science, University of Calabria, 87036 Rende, Italy; annafranca.farfalla@gmail.com (A.F.); giuseppe.cirillo@unical.it (G.C.); 8 Kids Cancer Centre, Sydney Children’s Hospital, Randwick, NSW 2052, Australia

**Keywords:** nanoarchitectures, gold nanoparticles, doxorubicin, DIPG, paediatric brain tumours, orthotopic animal models

## Abstract

**Simple Summary:**

Diffuse intrinsic pontine gliomas (DIPGs) are the most aggressive high-grade gliomas known to affect children. Due to the infiltrative nature of the DIPG tumours in the brainstem, they are very difficult to treat. Unfortunately, children succumb to their disease within 1–2 years from their diagnosis. Novel therapeutic treatments are, thus, urgently needed. Using primary cultures and orthotopic models of DIPG, we evaluated the therapeutic efficacy of passionfruit like nanoarchitectures functionalized with human serum albumin and loaded with doxorubicin (NA-HSA-Dox). We found that NA-HSA-Dox were significantly effective at penetrating DIPG spheroids and subsequently at reducing the proliferation and colony formation in DIPG cells. Although an antitumour effect was not observed in orthotopic models of DIPG, NA-HSA-Dox was well tolerated. These study demonstrates the importance of employing brain tumour orthotopic models for the identification of novel therapies and the need to develop treatments that effectively cross the blood brain barrier.

**Abstract:**

Diffuse Intrinsic Pontine Gliomas (DIPGs) are highly aggressive paediatric brain tumours. Currently, irradiation is the only standard treatment, but is palliative in nature and most patients die within 12 months of diagnosis. Novel therapeutic approaches are urgently needed for the treatment of this devastating disease. We have developed non-persistent gold nano-architectures (NAs) functionalised with human serum albumin (HSA) for the delivery of doxorubicin. Doxorubicin has been previously reported to be cytotoxic in DIPG cells. In this study, we have preclinically evaluated the cytotoxic efficacy of doxorubicin delivered through gold nanoarchitectures (NAs-HSA-Dox). We found that DIPG neurospheres were equally sensitive to doxorubicin and doxorubicin-loaded NAs. Colony formation assays demonstrated greater potency of NAs-HSA-Dox on colony formation compared to doxorubicin. Western blot analysis indicated increased apoptotic markers cleaved Parp, cleaved caspase 3 and phosphorylated H2AX in NAs-HSA-Dox treated DIPG neurospheres. Live cell content and confocal imaging demonstrated significantly higher uptake of NAs-HSA-Dox into DIPG neurospheres compared to doxorubicin alone. Despite the potency of the NAs in vitro, treatment of an orthotopic model of DIPG showed no antitumour effect. This disparate outcome may be due to the integrity of the blood-brain barrier and highlights the need to develop therapies to enhance penetration of drugs into DIPG.

## 1. Introduction

Diffuse intrinsic pontine gliomas (DIPGs) are the most aggressive high-grade gliomas known to affect children [1]. Conventional radiotherapy is the only currently available treatment option that improves neurologic function and survival, although ultimately is not curative [2]. Despite multiple clinical trials being conducted the prognosis has not improved with almost all children succumbing to the disease within 1–2 years from the diagnosis. Due to the infiltrative nature and location of this tumour, surgical resection is not possible; thus, novel chemotherapeutic treatment options are urgently needed [3,4]. The identification of key driver mutations on histone H3 (H3K27M) present in nearly 80% of the DIPG cases as well as other genomic alterations such as TP53, ACVR1, PI3K mutations and PDGFRa amplification has renewed the hope for the development of targeted therapies for this aggressive disease [5,6,7].

DIPGs are diagnosed by their typical appearance on magnetic resonance imaging (MRI). The lack of enhancement on T1 post-contrast images has been suggested to reflect an intact blood-brain barrier (BBB), which may be an important factor leading to the failure of potential anti-DIPG therapies [8]. We and others have shown that the BBB remains intact in preclinical models of DIPG [9,10,11]. The BBB is a unique and complex network of cells, which restricts the transport of toxic chemicals including chemotherapies from the circulation to the brain [12]. Various strategies are currently under development to enhance the delivery of therapies in the brain. Convection enhanced delivery (CED) which involves the administration of chemotherapeutic agents through the implantation of a catheter directly into the tumour site, thus bypassing the BBB is under Phase I clinical development for brain tumour patients including DIPG for various targeted agents and antibodies [4,13,14,15]. Methods that disrupt the BBB such as ultrasound directed microbubbles have also shown promising preclinical outcomes in brain tumour models [16,17,18].

Radiotherapy has also been implicated in enhancing the penetration of chemotherapy. Although the exact mechanism by which the BBB may become disrupted is not fully understood, a few studies have indicated that structural changes in the endothelial cells as well as decreased expression of efflux pumps can render it more penetrable [19,20]. Animals treated with irinotecan following irradiation were found to exhibit significantly higher levels of irinotecan in the cerebrospinal fluid [21]. Qin et al. reported an increase in methotrexate concentrations in cerebrospinal fluid of adult patients with gliomas [22].

The development of nanoparticles as Trojan horses for the delivery of chemotherapies across the BBB has been of great interest for many years for improving drug bioavailability, and penetration across the BBB and reducing systemic side effects [23,24]. Gold nanoparticles in particular have piqued interest due to many advantages including relatively simple production, peculiar light-matter interactions and easy surface modification with biomolecules and drugs [25,26]. However, few applications have been translated due to toxicity issues with long term accumulation in organs [27,28]. A phase I clinical trial is currently being conducted with gold-based nanoparticle NU-0129 functionalised with siRNA targeting the BCL2 gene in adult patients with glioblastoma (clinicaltrials.cov/NCT03020017). Other metal-based nanoparticle approaches such as gadolinium (NCT03818386), gold/silica (NCT0280535) are also currently under evaluation in adult malignancies.

Passionfruit-like nanoarchitectures (NAs) are nature-inspired nanoparticles that have demonstrated promising preclinical behaviours that warrants evaluation on the bench side [29,30]. NAs consist of (bio)degradable 100 nm silica capsules comprising non-persistent 3 nm gold nanoparticles in polymeric matrices [31]. NAs have demonstrated in vitro cytotoxicity in cancer cells, biodegradation within 48 h and adequate clearance from living vertebrates without systemic toxicity when loaded with various metals [32,33]. The double surface of the silica nanoarchitecture is associated to an impendence mismatch and were successfully employed for dual photoacoustic/ultrasound imaging application [29]. Furthermore, NAs comprise ultrasmall gold nanoparticles that can synergistically combine the chemotherapeutic action with light-matter interactions, as demonstrated in the treatment of head and neck squamous cell carcinomas [34]. Finally, coating NAs with organic molecules such as peptides or proteins has proved to be a valuable strategy for the fabrication of highly biocompatible multifunctional carriers for the vectorization of cytotoxic drugs to different cancer types [35,36,37].

In this study, we pre-clinically evaluated human serum albumin coated passion fruit-like nanoarchitectures (NAs-HSA) loaded with the chemotherapeutic agent doxorubicin (NAs-HSA-Dox). We show the cytotoxic and apoptotic activity of NAs-HSA-Dox in a panel of DIPG cultures and its ability to penetrate DIPG neurospheres.

## 2. Materials and Methods

### 2.1. Preparation of Nanoparticles

The synthesis of passionfruit-like nano-architectures (NAs) was performed by following our standard protocol and the product characterized by our characterization cascade assay [38]. Briefly, gold ultrasmall nanoparticles (USNPs) were prepared by adding in 20 mL of Milli-Q water 200 μL of aqueous solution of HAuCl_4_ (stock: 10 mg/mL) and 10 μL poly(sodium 4-styrene sulfonate) (70 kDa, 30% aqueous solution, PSS). The chloroauric solution was stirred vigorously and 200 μL of sodium borohydride (stock: 8 mg/mL) was quickly added. The solution was then stirred vigorously for 2 min and was aged for 10 min. Then, 75 μL of aqueous poly(L-Lysine) (stock: 40 mg/mL) was added for controlled aggregation of the PSS-coated Au USNPs. The solution was further incubated for 20 min. The Au polymeric aggregates were collected by centrifugation at 14,000× *g* rpm for 5 min and resuspended in 2 mL Milli-Q water. Then, a modified Stöber process was employed to allow silica shell formation in the periphery of Au polymeric aggregates. Two 50 mL tubes were filled each with 35 mL of ethanol, 1.2 mL ammonium hydroxide solution (30% in water), 20 μL tetraethyl orthosilicate (TEOS, 98%), and 1 mL of the Au polymeric aggregates. The reaction was allowed to proceed with moderate shaking for 3 h. Then, the tubes were centrifuged at 4000× *g* rpm for 30 min. After discarding the supernatant, the precipitates containing the nano-architectures (NAs) were washed by resuspending in ethanol, sonicating, and centrifugation at 14000× *g* rpm for 3 min. The washing was discarded and another round of re-suspension-sonication-centrifugation was performed. Then, short spin (15 s or until the rotational speed reaches 14,000× *g* rpm) was done to remove bigger NAs. The supernatant was spun again for 3 min at 14,000× *g* rpm to collect the nano-architectures. Finally, NAs were resuspended and stored in ethanol (1 mL). NAs surface were coated with HSA as follows: 1.0 mL of NAs solution (2 mg/mL) in ethanol was added to 4 mL HSA solution (2.5 mg/mL) in phosphate buffer (0.01 M, pH 7.4) and allowed to stand under stirring at 37 °C for 20 min. Thereafter, the reaction solution was centrifuged three times (2 min each at 13,400× *g* rpm) and the precipitate recovered in milli-Q water. The resulting solution was frozen and dried with a freeze drier (Micro Modulyo, Edwards Lifesciences, Irvine, CA, USA) to afford vaporous solids (NAs-HSA).

### 2.2. Drug Delivery Studies

DOX loading efficiency of nanoparticles (NAs-HSA or NAs) was determined by mixing 1.5 mg DOX with 15 mg nanoparticles in 10 mL distilled water under magnetic stirring at room temperature for 24 h. Then, samples were centrifuged for 2 min at 13,400× *g* rpm and the loading degree of nanoparticles was calculated based on the unbound amount of DOX detected in the collected supernatants according to the following Equation (1) [39]:

(1) $$LD\left(\%\right)=\frac{weight\;of\;encapsulated\;DOX\;\left(mg\right)}{total\;weight\;of\;DOX+nanoparticles\;\left(mg\right)}\times 100$$

For release and biological experiments, DOX loaded nanoparticles (NAs-HSA-Dox) were prepared using a loading degree of 2.7% by mixing 1.35 mg DOX with 48.65 mg NAs-HSA in 10.0 mL distilled water under magnetic stirring at room temperature for 24 h, and collecting the loaded sample (NAs-HSA-Dox) after freeze-drying procedure. An uncoated control (NAs-Dox) was composed by employing 48.65 NAs instead of NAs-HSA in the previous procedure. The extraction method was adopted for the evaluation of entrapped drug [40]. In separate experiments, 50 mg dried loaded NAs and NAs-HSA were immersed in 25 mL Acetate Buffer (0.1 mol/L, pH 5.0) in a dialysis bag (MWCO: 12,000–14,000 Da, VWR International GmbH, Darmstadt, Germany), and dialyzed against 100 mL of the same buffer. The buffer solution was replaced with an equal amount and the process was continued until no drug remained in the carrier. The buffer containing extracted drug was freeze-dryed, dissolved in water and analyzed by UV-Vis.

The DOX release profiles were recorded by immersing, in separate experiments, 15 mg NAs-HSA-Dox or NAs-Dox (0.41 mg Dox equivalent) into 1.5 mL releasing media at the selected pH (Phosphate Buffered Saline 0.01 mol/L, pH 7.4 or Acetate Buffer (0.1 mol/L, pH 5.0) in the dialysis bag, and dialyzed against 13.5 mL of the corresponding buffer. At predetermined time intervals, the cumulative amount of drug released was calculated using the following Equation (2):

(2) $$DOX\;release\;\left(\%\right)=\frac{M_{t}}{M_{0}}$$

where M_t_ and M_0_ are the amounts of drug in solution at time t and loaded into the carrier, respectively.

Analytical determination of DOX was performed by using an Evolution 201 spectrophotometer (ThermoFisher Scientific, Hillsboro, OR, USA) and the calibration curves of DOX sketched in the selected buffer (pH 7.4 or pH 5.0).

To study the release mechanism, four different kinetic models were applied to fit the experimental data [41].

Model 1 is the zero order kinetic and is expressed by the following Equation (3):

(3) $$\frac{M_{t}}{M_{0}}=kt$$

where *k* is the kinetic constrant and *t* the time of release.

Model 2 represent the first order kinetic (Equation (4)):

(4) $$\frac{M_{t}}{M_{0}}=1-e^{-kt}$$

*k* is the kinetic constant and *t* the time of the release, *a* is the release coefficient.

Model 3 is given by the Ritger–Peppas Equation (5):

(5) $$\frac{M_{t}}{M_{0}}=kt^{n}$$

where *k*_1_ is the kinetic constrant, *t* the time of the release constant, and *n* is the coefficient indicatin g the mechanism of the release. *n* ≤ 0.43 indicates a Fickian diffusion mechanism; *n* = 0.84 a Case II transport, and 0.43 *n* < 0.85 anomalous stransport mechanism.

Model 4 is described by the Peppas–Sahlin Equation (6):

(6) $$\frac{M_{t}}{M_{0}}=k_{1}t^{1/2}+k_{2}t$$

*k*_1_ and *k*_2_ are the kinetic constant of Fickian and anomalous diffusion, respectively.

All chemicals were purchased from Merck KGaA (Darmstadt, Germany).

The studies conducted herein, including material characterisation, biological characterisation, and experimental details, conform to the Minimum Information Reporting in Bio–Nano Experimental Literature MIRIBEL reporting standard for bio–nano research [42], and we include a companion checklist of these components in the Supplementary Information.

### 2.3. Transmission Electron Microscopy

Transmission electron microscopy (TEM) observations were carried out with a ZEISS Libra 120 TEM (Zeiss, Oberkochen, Germany) operating at an accelerating voltage of 120 kV. The samples were deposited on 300-mesh copper TEM grids coated with a carbon film. The depositions were performed by immersion of the grid into the colloidal solution. Imaging was carried out after the evaporation of the solvent. Hydrodynamic diameter and zeta potential measurements were performed on Malvern Zetasizer nano ZS90 (Malvern Instruments, Malvern, UK) using, respectively, standard cuvettes and DTS 1060 capillary cell. During measurements, the nanoparticles were resuspended in PBS (2 mL) at pH 7.4 and sonicated for 5 min. The reported values are average of five consecutive measurements [43].

### 2.4. Human DIPG Neurosphere-Forming Cultures

Primary DIPG cultures were grown mainly as neurospheres in all the experiments listed in this study. All cultures have been validated by STR profiling and mycoplasma testing. DIPG cells were use within the following passage ranges HSJD-DIPG007—p36–37, SU-DIPGVI—p29-p41, RA055—P2-P5 and VUMC-DIPG10—P3-P6. DIPG cultures were grown in stem cell media containing half quantity of DMEM/F12 and half of the Neurobasal medium (Invitrogen, ThermoFisher Scientific, Waltham, MA, USA) supplemented with glutamax, pyruvate, non-essential amino acids, HEPES buffer, antibiotic/antimycotic (Invitrogen). Stem cell media were freshly supplemented each time with heparin (Stem Cell Technologies, Vancouver, BC, Canada), human EGF, human basic FGF, PDGF-AA and PDGF-BB (Stem Cell Technologies) [44]. Cultures were maintained at 37° C in a humidified atmosphere with 5% CO_2_.

### 2.5. Proliferation Assays

DIPG cells were plated at a cell density of 2000–3000 cells/well in flat bottom 96 well plates and incubated for 72 h. Cells were subsequently treated for 72 h with NA-HSA, free-doxorubicin and NA-HSA loaded with doxorubicin over a range of equivalent doxorubicin concentrations. Sensitivity to each agent was assessed with the alamar blue assay (Sigma-Aldrich, Castle Hill, NSW, Australia) and results are presented as percentage viability compared to untreated cells.

### 2.6. Soft Agar Colony Assays

Soft agar assays were performed to assess the effects of NA-HSA, free-doxorubicin and NA-HSA-Dox in the clonogenic ability of the DIPG cells. The assay was performed in 24-well plates as previously described [45]. Briefly in each well, 400 μL of 0.5% agar (in stem cell culture medium) was added as the bottom layer followed by 300 μL of 0.3% agar as the top layer. Approximately 800 HSJD-DIPG007 and RA055 cells were added in the top layer and treated with indicated doses. DIPG colonies were maintained at 37 °C in a humidified 5% CO_2_ atmosphere for two–three weeks. The colonies were counted using MTT and presented as percentage colony formation compared to untreated.

### 2.7. Western Blot Experiments

HSJD-DIPG007 and RA055 cells were plated at a cell density of 0.5–1 million cells and allowed to grow as spheroids. Subsequently cells were treated with Dox, NAs-HSA, and NAs-HSA-Dox at indicated doses for 24 h. Following treatments cells were washed with PBS and proteins were extracted using the RIPA Buffer according to manufacturer protocol, (Sigma-Aldrich). Protein content was subsequently quantified with the BCA Protein Acid Assay Kit (Pierce, ThermoFisher Scientific, Waltham, MA, USA). Proteins were resolved on 12–20% Tris-HCl SDS-PAGE precast gels (Bio-Rad, Gladesville, NSW, Australia) and blotted as described by manufacturer’s instructions (Bio-Rad). DIPG cell lysates were analysed with the following antibodies overnight at 4 °C; cleaved caspase 3 (1:1000, #9664S), cleaved parp (1:1000, #5625S), P-H2AX (1:1000, #9718S), GAPDH (1:2000, #2118), beta-actin (1:2000, #4970) while anti-rabbit HRP-linked monoclonal antibody was used as a secondary antibody for 2 h at room temperature (1:2000, #7074). All antibodies were purchased from Cell Signalling (Danvers, MA, USA).

### 2.8. High Content Imaging

HSJD-DIPG007 cells were plated in 96-well U-bottom plates in 100 μL of stem cell media at the cell density of 6000 cells/well. A total of three days following incubation, single spheroids formed at the bottom of each well. Each spheroid was treated in duplicate with NAs-HSA-Dox containing equivalent doses of 1, 2 and 4 μM of doxorubicin for 24 h. Cells were washed once with PBS and stained with Hoechst 33342 (Invitrogen). Spheroids were imaged using the Operetta high-content imager at 10 X magnification and acquiring a stack of 7 separate images (at 20 μm intervals) for each well/spheroid. An image analysis workflow was developed to segment the spheroids using the Hoechst fluorescence readout, and then this was used to quantitate the fluorescence intensity for doxorubicin in the spheroid. The mean fluorescence intensity for doxorubicin in each spheroid was calculated, and this was used as the final readout for doxorubicin uptake in each treatment condition.

### 2.9. Confocal Imaging

Optical sections of each spheroid (100 μm from the bottom of the spheroid) were imaged on a Zeiss LSM 880 (Zeiss, Oberkochen, Germany) using multiphoton excitation. Images were analysed in Fiji Image J [46] by drawing 10 random lines from the edges through the centre of each spheroid and plotting intensity profiles along these lines. A total of six spheroids each from two independent experiments were analysed for Dox, and NAs-HSA-Dox treated spheroids, and four spheroids from two independent experiments were analysed for NAs-HSA treated spheroids. Statistical analysis was carried out using GraphPad Prism (GraphPad Software, San Diego, CA, USA).

### 2.10. Orthotopic DIPG Animal Model and Drug Treatments

Pathogen-free, 5–7-week-old female Balbc/Nude mice were purchased from Animal Resources Center (Perth, Australia) and kept at an ambient temperature of 22 °C under a 12-h light cycle (7:00 a.m.–7:00 p.m.). HSJD-DIPG007 cells (200,000 cells) were re-suspended in 2 μL of matrigel and injected intracranially into the brainstem of mice by using a stereotactic device (Kopf Instruments, Tujunga, CA, USA) (coordinates X = 0.5 mm Y = −6.0 mm, Z = −3.5 mm, with the bregma being used as a reference point). Treatments commenced at four weeks post intracranial injection. Animals were injected intravenously with saline, NAs-HSA (50 mg/kg/day) NAs-HSA loaded with an equivalent dose of doxorubicin (1.35 mg/kg/day) and doxorubicin (1.35 mg/kg/day) intravenously 3 days per week (Monday, Wednesday, Friday) for 3 weeks. Mice exhibiting neurologic decline such as ataxia, head tilting with or without 20% weight loss were humanely euthanased for histological analysis of brainstem tumours. Three animals were humanely euthanased after the last treatment for determination of gold levels in the brains 3 h after the last treatment while two animals per cohort were euthanased for immunohistochemistry. Brains were fixed in 10% with formalin neutral buffered solution (Sigma-Aldrich, Castle Hill, NSW, Australia) embedded in paraffin wax and 5-mm sections were cut and mounted on glass slides. Following dehydration, sections were stained with hematoxylin/eosin and Ki67 for histologic examination. All animal experiments were performed according to the Australian Code of Practice for the Care and Use of Animals for Scientific Purposes under the Animal Research Regulation of the New South Wales (Australia) and under a protocol approved by the Animal Use and Care Committees of University of New South Wales.

### 2.11. Detection of Gold in DIPG Xenografted Brains

Following the last treatments, mice were humanely euthanased, and brains were harvested and were immediately snap-frozen. Subsequently brain tissue samples were lysed with 1ml of 70% w/w nitric acid. Lysates were diluted further with miliQ water and analysed for the presence of gold atoms using an Elan 6100 Inductively Coupled Plasma Mass Spectrometer (ICP-MS)(Perkin Elmer, Waltham, MA, USA).

### 2.12. Statistical Analysis

Data were analysed with GraphPad Prism using an unpaired Student t-test. All tests are two-tailed. P values less than 0.05 were considered to be statistically significant. Results have been displayed as average ± SEM. The t-test (Two-sample assuming equal variances) was applied to the evaluation of loading degree of NAs and NAs-HSA. Statistical significance between survival curves was analysed using the Mantel-Cox with GraphPad Prism (GraphPad Software, San Diego, CA, USA).

## 3. Results

### 3.1. Preparation and Characterization of NAs-HSA-Dox

**Figure 1:**
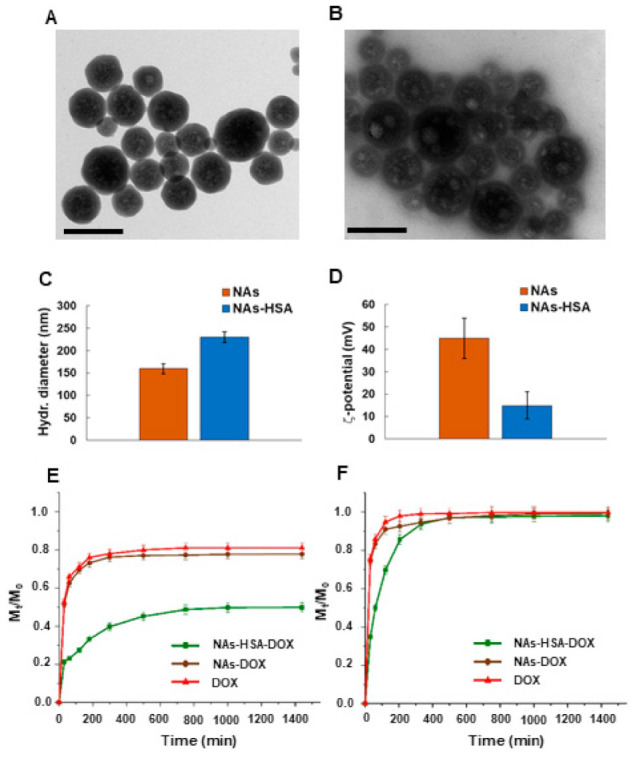
Characterization of Nanoparticles; TEM analysis of (A) NAs and (B) NAs-HSA (scale bar 200 nm); DLS measurements for the determination of (C) size and (D) zeta potential of NAs and NAs-HSA. pH responsive DOX release profile (M_t_/M_0_) from NAs-HSA (■), NAs (●) and DOX solution (▲) at (E) pH 7.4 and (F) pH 5.0 recorded by UV-Vis spectroscopy. The effective coating of NAs by HSA did not affect the spherical shape of the nano-architectures, while resulting in a hydrodynamic diameter increase from 160 to 230 nm and zeta potential decrease from −45 to −15 mV. NAs-HSA were able to selectively vectorize drug molecules to acidic environments.

Effective HSA coating of passionfruit-like nanoarchitectures was confirmed by combined dynamic light scattering and TEM analysis (Figure 1). The presence of the protein coating did not affect the spherical shape of the nano-architectures, while it modified the surface properties, with the hydrodynamic diameter increasing from 160 to 230 nm and the zeta potential decreasing from −45 to −15 mV.

The presence of USNPs within NAs was shown previously [47]. Here, the differences in the TEM and DLS measurement confirm the successful decoration of NAs with HSA without affecting the structure of the final nano-architectures. Indeed, by TEM measurement we are able to observe the structures with many electrons (for example, silica, gold etc.). Thus, the decoration with proteins on the surface of NAs cannot be observed. On the other hand, DLS measurements collect the hydrodynamic diameter, that is affected by the surface features of the nanomaterials. In this case, the hydrodynamic diameter increase is directly correlated to the grafting of HSA on NAs.

The doxorubicin release profile from NAs-HSA-Dox, depicted in Figure 1E,F, revealed the ability of the synthesized nanocarrier to release the payload in response to the variation of the environmental pH. Since nanoparticle possessed different drug loading degree (9% and 3.5% for NAs-HSA and NAs, respectively) due to presence/absence of the HSA coating [48,49], experiments were performed by selecting a soaking/freeze drying procedure and a LD value of 2.7% to not exceed the LD of NAs and ensure that the same amount of Dox is loaded into the two carrier samples. Experimental evidence proving this statement was obtained by extraction method according to the literature [40], obtaining no significant differences in the recorded LD values for the two samples (*p* > 0.05).

The doxorubicin release was found to be higher and faster at acidic pH vs. physiological pH: an almost complete release was recorded at pH 5.0 after 5 h, while only 50% of loaded drug was released at pH 7.4 after 24 h. The doxorubicin releasing profile of NAs-HSA-Dox was compared to the uncoated control (NAs-Dox), confirming that the presence of HSA maximizes the drug-to-carrier interaction. Indeed, NAs-Dox showed higher amount of release at both pH values, with the value recorded at physiological pH after 24 h incubation being close to 80%.

To further confirm this statement, the loading profiles were compared to the diffusion of the free drug in the two pH conditions and analysed by four different kinetic models. The best fit model of the experimental data for each sample/pH value was assessed by comparing the R^2^ values (Table S1). By analyzing the kinetic parameters of each model, the influence of HSA coating in the modulation of the DOX release is evident. The diffusion of free DOX in the surrounding environment, indeed, followed a first order kinetics at both pH values, being almost unmodified after loading into NAs due to the poor drug-to-carrier affinity. A totally different behavior is observed in the case of NAs-HSA-Dox, where a Ritger–Peppas model can be applied indicating that the drug release is a combination of Fickian and non-Fickian (anomalous) diffusion [43]. Furthermore, the influence of pH on the DOX release can be highlighted by considering the *n* value of Ritger–Peppas model and applying the Peppas–Sahlin equation, where the Fickian and the anomalous contributions to the drug release are simultaneously quantified by the first (k_1_) and the second (k_2_) term, respectively [50]. It is evident that the DOX is released mainly via a Fickian diffusion at pH 7.4 (*n <* 0.43 and k_2_/k_1_ = 79.5) and that at acidic pH the release rate increased, with the anomalous contribution being more effective in influencing the drug release (*n* = 0.45 and k_1_/k_2_ reduced to 52.9) as a consequence of the DOX protonation and the different drug-to-carrier interactions.

### 3.2. Cytotoxic Efficacy of Doxorubicin-Loaded Gold Nanoparticles in Neurosphere-Forming DIPG Cultures

**Figure 2:**
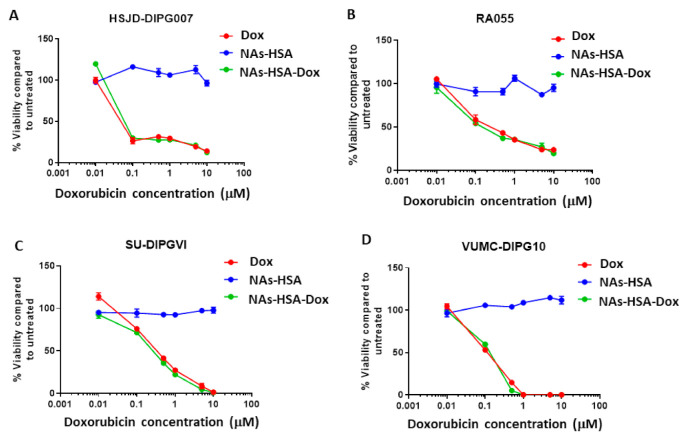
Gold nanoparticles loaded with doxorubicin reduce cell proliferation in DIPG neurospheres; (**A**–**C**) Cytotoxic efficacy of doxorubicin (Dox), Gold nanoparticles (NAs-HSA) and NAs-HSA loaded with doxorubicin (NAs-HSA-Dox) for 72 h in H3K27M DIPG cultures HSJD-DIPG007, RA055 and SU-DIPG-VI; (**D**) Cytotoxic efficacy of Dox, NAs-HSA and NAs-HSA-Dox for 72 h in H3K27wt DIPG culture VUMC-DIPG-10. Alamar blue assay was used to assess viability, presented as % viability compared to untreated cells. Experiment was performed twice, N = 8 each time.

Doxorubicin has been employed in multiple studies for its ability to induce cytotoxic effects in gliomas, including DIPG. Despite its potent antitumour efficacy in multiple cancers, this drug has limited therapeutic efficacy in brain tumours [17,51]. Using the alamar blue based cell proliferation assay, we investigated the cytotoxic efficacy of doxorubicin in a panel of neurosphere forming DIPG cells containing either H3K27M (HSJD-DIPG007, RA055, SU-DIPGVI) or H3K27 wild type (VUMC-DIPG10) and compared it to NAs-HSA and NAs-HSA loaded with doxorubicin (NAs-HSA-Dox). The use of 3D tumour models is thought to more accurately reflect the clinical scenario due to their close representation of physiological tumour conditions [52]. All four primary DIPG cultures exhibited similar cytotoxic efficacy between doxorubicin and NAs-HSA-Dox. In contrast, naked NAs-HSA did not show any cytotoxic effects (Figure 2). The response to each agent was similar among the H3K27M-containing (Figure 2A–C) cultures and H3K27wt (Figure 2D).

**Figure 3:**
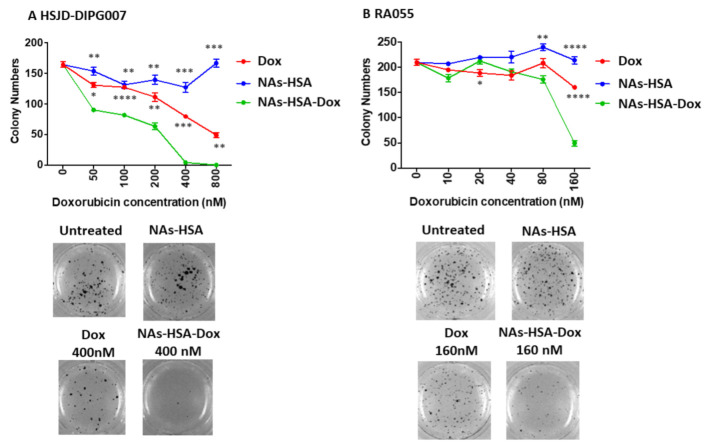
DIPG colony formation is reduced upon treatment with doxorubicin-loaded gold nanoparticles; (**A**/**B**) Soft agar colony assays were performed in two DIPG primary cultures (**A**) HSJD-DIPG007 and (**B**) RA055 DIPG cells with various concentrations of Dox, NAs-HSA-Dox and NAs-HSA. Colonies were stained with MTT after 2–3 weeks and counted. Both cultures display significantly lower colony formation when treated with NAs-HSA-Dox compared to doxorubicin alone. In contrast, DIPG cells treated with NAs-HSA had no effects on colony formation. * *p* < 0.05; ** *p* < 0.01; *** *p* < 0.001; **** *p* < 0.0001. The experiment was performed twice N = 4 each time.

To further investigate the anti-DIPG potential of NAs-HSA-Dox, we performed anchorage-independent colony formation assay in two H3K27M DIPG cultures (Figure 3). We found the clonogenic potential of HSJD-DIPG007 and RA055 cells was significantly reduced in NAs-HSA-Dox treated cells compared to cells treated with the same amount of free doxorubicin. Similarly to cytotoxic assays, we did not observe any effects mediated by the empty NAs-HSA. These data demonstrate that the anti-clonogenic and anti-proliferative activity of doxorubicin is significantly enhanced when it is delivered though NAs-HSA.

**Figure 4:**
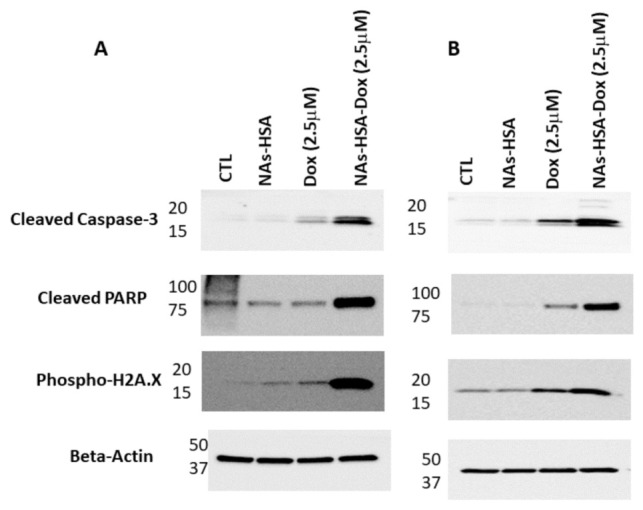
NAs-HSA-Dox enhances apoptotic cell death in DIPG neurospheres; (**A**) HSJD-DIPG007 cells were treated with NAs-HSA-Dox and free Dox at the equivalent dose of 2.5 μM for 24 h; (**B**) RA055 cells were treated with NAs-HSA-Dox and free Dox at the equivalent dose of 2.5 μM for 24 h; Representative images of western blots show elevated levels of apoptotic markers cleaved-caspase 3 and cleaved-PARP in the NAs-HSA-Dox-treated cells. Similarly, an increase in Phospho-H2A.X was observed in NAs-HSA-Dox-treated DIPG cells. In contrast no difference was observed among the untreated, NAs-HSA and free Dox treated samples. Western blot analysis was performed twice for each primary DIPG culture (Figure S1).

We next sought to examine whether NAs-HSA-Dox enhances apoptotic cell death in HSJD-DIPG007 and RA055 cultures. Using immunoblot analysis, we found higher protein levels of cleaved-parp and cleaved-caspase 3 in NAs-HSA-Dox treated DIPG cells compared to cells treated with the same amount of free doxorubicin (Figure 4A,B). In contrast untreated and NAs-HSA treated cells did not influence levels of these apoptotic markers. Furthermore we also tested for the induction of Phospho-H2AX, which is an early sign of cell cycle arrest. Similarly we saw an increase in phosphorylation of H2AX in NAs-HSA-Dox treated DIPG cells compared to free doxorubicin (Figure 4A,B, Figure S1A,B, Figure S2A,B, Figure S3A,B). These results further support the capability of these gold nanoparticles to potently enhance the cytotoxic and apoptotic ability of doxorubicin.

### 3.3. Penetration of Doxorubicin-Loaded Nanoparticles in DIPG Neurospheres

**Figure 5:**
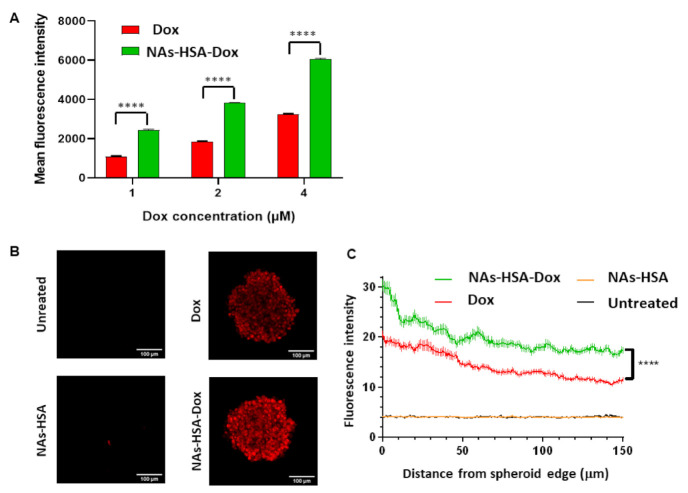
Penetration of NAs-HSA-Dox in DIPG neurospheres; (**A**) High-content imaging of HSJD-DIPG007 spheroids treated for 24 h with NAs-HSA-Dox and free Dox at equivalent doses of 1, 2 and 4 μM. Significantly higher uptake of NAs-HSA-Dox was observed among all the doses tested compared to free Dox (**** *p* < 0.0001). Bar represents 100 μm; (**B**) Representative optical sections of HSJD-DIPG007 spheroids exposed to 4 μM dox or NAs-HSA-Dox for 24 h; Experiment was performed once N = 6; (**C**) Fluorescence intensity profiles from edges of HSJD-DIPG007 spheroids. Significantly higher intensity is observed of Dox in NAs-HSA-Dox treated samples compared to free Dox (**** *p* < 0.0001 two way Anova) Experiment was performed three times N = 3.

Having established that NAs-HSA-Dox exhibits higher cytotoxicity and apoptotic cell death compared to free doxorubicin, we sought to understand whether the reason for this efficacy was enhanced penetration into the DIPG neurospheres. We firstly used high content imaging to evaluate the uptake of doxorubicin, by measuring fluorescence intensity using the Operetta Imager. We found that with increasing concentrations of Dox the intensity of fluorescence was higher for free Dox and NAs-HSA-Dox treated spheroids (Figure 5A). Furthermore fluorescence intensity was significantly higher in NAs-HSA-Dox treated samples compared to Dox free samples (Figure 5A).

To evaluate further the penetration of NAs-HSA-Dox in DIPG spheroids, we performed confocal imaging at the highest concentration (4 μM). Following a 24 h treatment we visualised penetration of doxorubicin into DIPG spheroids, which is consistent with previously published studies in other cancers [53]. Both free Dox and NAs-HSA-Dox penetrated to the core of the spheroid. However, NAs-HSA–Dox penetration into DIPG neurospheres was significantly higher (Figure 5B,C).

### 3.4. In Vivo Efficacy of Doxorubicin-Loaded Nanoparticles in an Orthotopic Patient-Derived DIPG Animal Model

**Figure 6:**
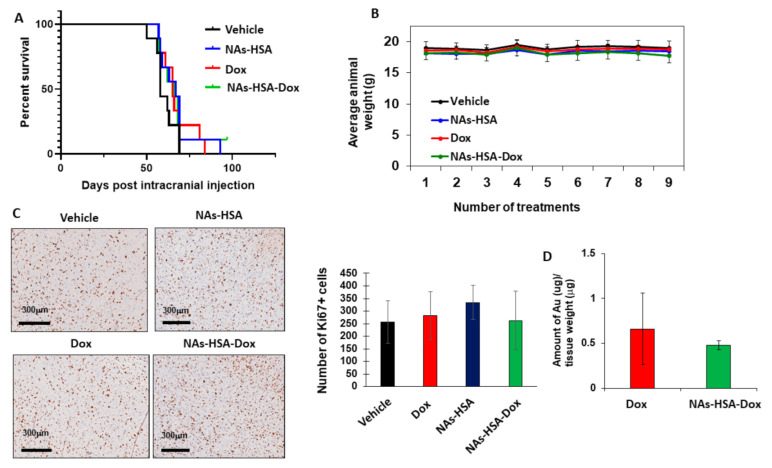
Therapeutic efficacy of gold nanoparticles (Au NP) loaded with Doxorubicin (Dox) in the HSJD-DIPG007 orthotopic animal model; (**A**) Survival curve of orthotopically-injected HSJD-DIPG007 into animals and subsequently treated with vehicle (0.9% Saline IV administration), NAs-HSA (50 mg/kg/day 3 days/week 3 weeks IV), Dox (1.35 mg/kg/day 3 days/week 3 weeks IV) and Nas-HSA loaded with Dox (equivalent to 1.35 mg/kg/day 3 days/week 3 weeks IV). Each treatment group consisted of 9 animals and tumour engraftment confirmed by neurological symptoms and/or weight loss. Therapeutic efficacy was not observed for any of the treatment cohorts (Vehicle vs. Dox *p* = 0.1421, Vehicle vs. Nas-HSA *p* = 0.1626, Vehicle vs. Nas-HSA-Dox *p* = 0.3932, Dox vs. Nas-HSA-Dox *p* = 0.9212); (**B**) Weight maintenance was similar among the four treatment cohorts for the duration of 9 doses (3 doses/week for 3 weeks); (**C**) Histologic analysis with Ki67 was performed on brains of two animals per cohort at the end of the treatments. Mostly, no differences were observed on Ki67 positive cells among the four treatments groups (Dox vs. Nas-HSA-Dox *p* = 0.188, Nas-HSA vs. Nas-HSA-Dox *p* = 0.321, Dox vs. Nas-HSA *p* = 0.420, Vehicle vs. Nas-HSA-Dox *p* = 0.113, Vehicle vs. Dox *p* = 0.063, Vehicle vs. Nas-HSA *p* = 0.032); Bar represents 300 μm; (**D**) Low levels of gold were identified in tumour-bearing animals treated with Nas-HSA-Dox (*p* = 0.368) 3 animals were used per treatment cohort at the end of the treatment.

Having demonstrated the cytotoxic and apoptotic potential of NAs-HSA-Dox in primary cultures of DIPG, we next sought to extend these findings in an orthotopic model of DIPG. The HSJD-DIPG007 orthotopic model has been demonstrated to mimic the clinical outcomes of this disease. DIPG neurospheres are injected in the 4th ventricle using a stereotactic device leading subsequently to infiltration of DIPG cells in the brainstem and cerebellum regions of the brain. Engrafted animals display progressive neurological decline such as head tilting, ataxia and weight loss [45,54]. Approximately 4 weeks past intracranial injection of DIPG cells, animals were treated with Dox 1.35 mg/kg/day intravenously for 3 days per week for 3 weeks. Another cohort received NAs-HSA-Dox (containing equivalent dose of 1.35 mg/kg/day of Dox) intravenously 3 days per week for 3 weeks. There were two groups—one receiving saline and one receiving empty NAs-HSA using the same treatment schedule for a total period of 3 weeks. During the treatment period we did not observe any significant toxicity in any treatment group apart from some scarring at the point of injection for Dox-only treated animals. Overall, the weights of the animals were maintained among all cohorts for the entire duration of treatment (Figure 6A). Following treatments we observed no significant changes in the median survival of the mice. Compared to saline-treated animals, there was no enhanced survival for the Dox-treated, and NAs-HSA-Dox treated animals (Figure 6B). Similarly to vehicle no changes were observed in median survival time for NAs-HSA treated animals (Figure 6B). Furthermore, histological analysis of brains showed infiltration of DIPG cells in the brainstem and cerebellum of mice while proliferation index marker Ki67 showed no significant differences among all treatment groups (Figure 6C). Since an intact blood-brain barrier has been considered the main limitation for the successful delivery of any therapy for diffuse gliomas, we determined the presence of gold atoms in the brains of animals engrafted with DIPG cells (Figure 6D). Using Inductively Coupled Mass Spectrometry (ICP-MS) we found the levels of gold atoms were at a very low level as indicating that lack of therapeutic efficacy may be due to inefficient penetration into the brain of engrafted animals (Figure 6D).

## 4. Discussion

DIPG is one of the most devastating brain tumours to affect young children. No systemic chemotherapy nor targeted therapy has shown to have any clinical impact. One of the key reasons for the failure of these systemic therapies is thought to relate to the intact blood-brain-barrier observed both in preclinical models and patients with DIPG [9,10]. There is an increasing interest in the development of new delivery methods in an attempt to improve the therapeutic efficacy of clinically available drugs and new targeted agents. In this study, we evaluated the therapeutic efficacy of doxorubicin carried by a new class of non-persistent gold-based Nas functionalized with HSA. We used a novel gold-based nanoparticle for the evaluation of doxorubicin efficacy when carried by Nas-HSA in preclinical models of DIPG. Nas are non-persistent metal-based nano-architectures with increasing potential in oncology and infectious disease management [29,30,32]. Nas have been developed with the main aim to translate plasmonic noble metals to the clinical practice in order to unlock their potential for innovative cancer treatments. Prior to performing any efficacy assessments, we extensively investigated their nanotoxicological and biokinetic profile in vertebrate models following injection and inhalation. We observed a very promising biosafety profile in zebrafish models [55], and confirmed these data further in healthy murine models [38]. Furthermore, we investigated the biokinetics of standard Nas in animal models and confirmed that the building blocks are metabolized or excreted after biodegradation [47]. Finally, it is important to highlight that the hydrodynamic diameter of Nas is not significantly altered over 8 h incubation in blood-mimicking solutions [33].

In this study, Nas were modified by a HSA coating, enhancing their affinity for doxorubicin without interfering with their dispersion properties, as demonstrated by a zeta potential value of around 20 mV being predictive of good short-term stability [56,57].

Our experiments demonstrated a superior doxorubicin release profile at low pH when Nas were functionalized with HSA, due to the ability of the protein to confer high affinity for drug molecules [58,59,60,61]. The recorded pH responsivity allowed hypothesizing an effective vectorization of the cytotoxic drug to the tumour site, with a reduction of the toxic side effects due to the low release in healthy tissues [62,63,64]. From a mechanistic point of view, literature data suggest that the release occurred as a consequence of the protonation of doxorubicin molecules at acidic pH, with a reduction of drug to carrier interactions, mainly consisting in hydrogen bonding, Van der Waals forces, and hydrophobic interactions. On the contrary, at high pH value, doxorubicin is deprotonated, resulting in hydrophobic property and affecting loading capacity [65,66].

We demonstrated similar cytotoxicity between Nas-HSA-Dox and free doxorubicin in DIPG cells in short term assays; however NA-HSA-Dox was significantly more effective at reducing colony formation and inducing apoptotic cell death. Doxorubicin on its own has been previously reported to be cytotoxic in DIPG at low micromolar concentrations; however, it is known to exhibit very poor BBB permeability [17], which is the reason that it has not been considered a therapeutic option for DIPG patients. Although H3K27M containing DIPG tumours are considered more aggressive than H3wt, we observed that VUMC-DIPG10 culture (H3K27wt) was slightly more sensitive to doxorubicin treatment than the other cultures (H3K27M) tested. A variety of approaches have been employed to enhance its penetration through the BBB, including focused ultrasound (FUS) delivery, gold nanoparticles and lipid-based nanoparticles with some exhibiting promising permeability outcomes [67]. Although enhanced survival in GBM orthotopic models has been observed with some doxorubicin nanoparticles, they have not previously been tested in DIPG models [68].

Despite having observed superior uptake of Nas-HSA-Dox compared to free-DOX in DIPG cells, no therapeutic benefit was demonstrated in orthotopic models of DIPG. Potentially this could be attributed to the limited penetration of Nas-HSA-Dox through the BBB as determined by the low levels of gold in the animals’ brains. This could have caused suboptimal concentrations of doxorubicin in the tumour. However, the gold levels were only determined after 3 h of intravenous administration of Nas-HSA-Dox, which could potentially be an insufficient period to determine the nanoparticle levels in the brain. Further studies are needed to perform in-depth pharmacokinetic analysis of Nas-HSA-Dox over different timepoints ranging between 30 min–24 h in order to understand the accumulation and clearance rates of these nanoparticles in brain tumours.

A further limitation of our study is the absence of pharmacokinetic analysis of doxorubicin in the brain. As discussed above, the Nas-has-Dox can release doxorubicin rapidly at low pH. Recent studies have indicated that DIPG tumours display unique interactions with neurons which further assist in tumour cell proliferation [69]. This electrical activity appears to be mediated through AMPA receptors [70]. However, this electrical activity has also been found to be independent of the presence of a neuronal network and be influenced by extracellular low pH changes mediated by the glioma cells [71]. It is possible in the presence of DIPG tumour cells that the low pH levels of the extracellular matrix mediate the release of doxorubicin before entry into the brain tumour. Further studies are needed to understand how the unique and complex extracellular environment of the brain tumours may influence the penetration and drug release of these nanoparticles. It would also be worth noting that other administration pathways, such as intranasal delivery, may result in an improved effect due to the enhanced transient accumulation of NAs in the brain [47]. Additionally, the employment of focused ultrasound together with chemotherapy-loaded liposomes has shown some promise in glioblastoma bearing mice [72,73]. However large-size liposomes (>120 nm) still remain a challenge even with FUS-mediated delivery into the brain [74]. A similar approach could potentially be employed in the future for the NAs studied here. Indeed, the penetration of chemotherapy-loaded gold nanoclusters together with FUS has been recently demonstrated in the brainstem region of healthy animals [75]. Overall, despite this preclinical therapeutic outcome, NA-HSA and NA-HSA-Dox nanoparticles have demonstrated a promising safety profile with animals exhibiting no weight loss or tissue scarring in contrast to free-Dox. DIPG tumours are regarded as the most chemoresistant brain tumours with many preclinical studies demonstrating a lack of therapeutic efficacy of many small molecule inhibitors [45,76]. The presence of multi-drug resistance proteins is a further limitation for the successful treatment of these aggressive tumours with chemotherapy [77]. Further studies are warranted to explore the pharmacokinetics of NA-HSA in healthy animals and tumour bearing mice as well as the antitumour efficacy with chemotherapeutic drugs or targeted agents exhibiting more potent, nanomolar efficacy in DIPG cells.

## 5. Conclusions

Our findings demonstrate promising cytotoxic activity of doxorubicin loaded nanoarchitectures against DIPG cells and superior uptake compared to free doxorubicin. Further studies are required to understand the pharmacokinetic profile of the NAs-HSA in orthotopic models of DIPG as well their efficacy when loaded with more effective chemotherapeutic agents or in combination radiotherapy.

## Data Availability

The data presented in this study are available in this article (and Supplementary Material).

## Supplementary Materials

The following are available online at https://www.mdpi.com/2072-6694/13/6/1278/s1, Figure S1: NAs-HSA-Dox enhances apoptotic cell death in DIPG neurospheres; Figure S2: Original western blots for Figure 4; Figure S3: Original Western blots for Figure S1; Table S1: Kinetic parameteters for DOX release obtained by applying the four kinetic models. All authors have read and agreed to the published version of the manuscript.
