# Supplementary material for: Doxorubicin-Loaded Gold Nanoarchitectures as a Therapeutic Strategy against Diffuse Intrinsic Pontine Glioma
Source: Cancers (Basel). 2021 Mar 13;13(6):1278. doi: 10.3390/cancers13061278 (PMC7999568; doi:10.3390/cancers13061278)
Supplement: Supplementary file 1 [file cancers-13-01278-s001.pdf]

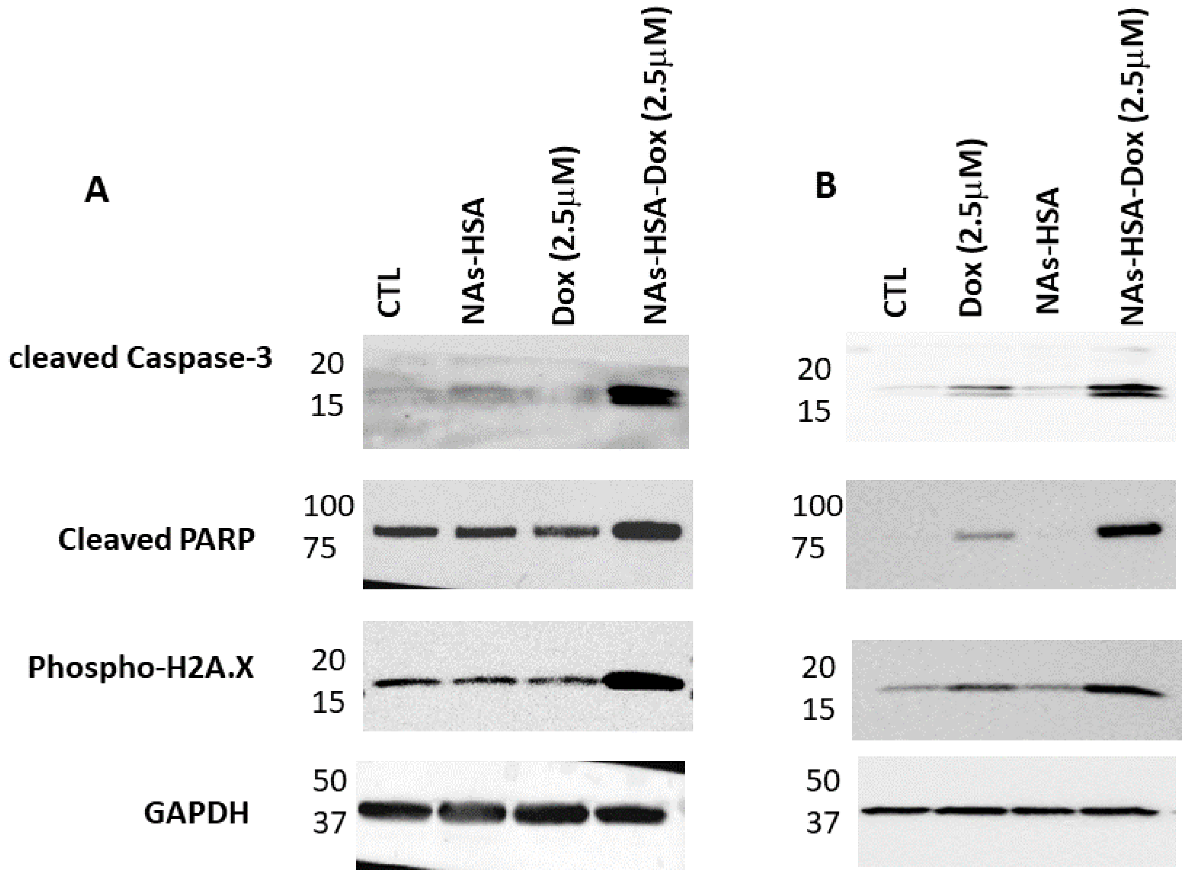

Figure S1: NAs-HSA-Dox enhances apoptotic cell death in DIPG neurospheres; Similarly to Figure 4 (A) HSJD-DIPG007 cells were treated with NAs-HSA-Dox and free Dox at the equivalent dose of 2.5 μM for 24h; (B) RA055 cells were treated with NAs-HSA-Dox and free Dox at the equivalent dose of 2.5 μM for 24h; Similarly to Figure 5 we observed increased levels of cleaved-caspase 3, cleaved-PARP and phospho-H2A.X in the NAs-HSA-Dox-treated cells.

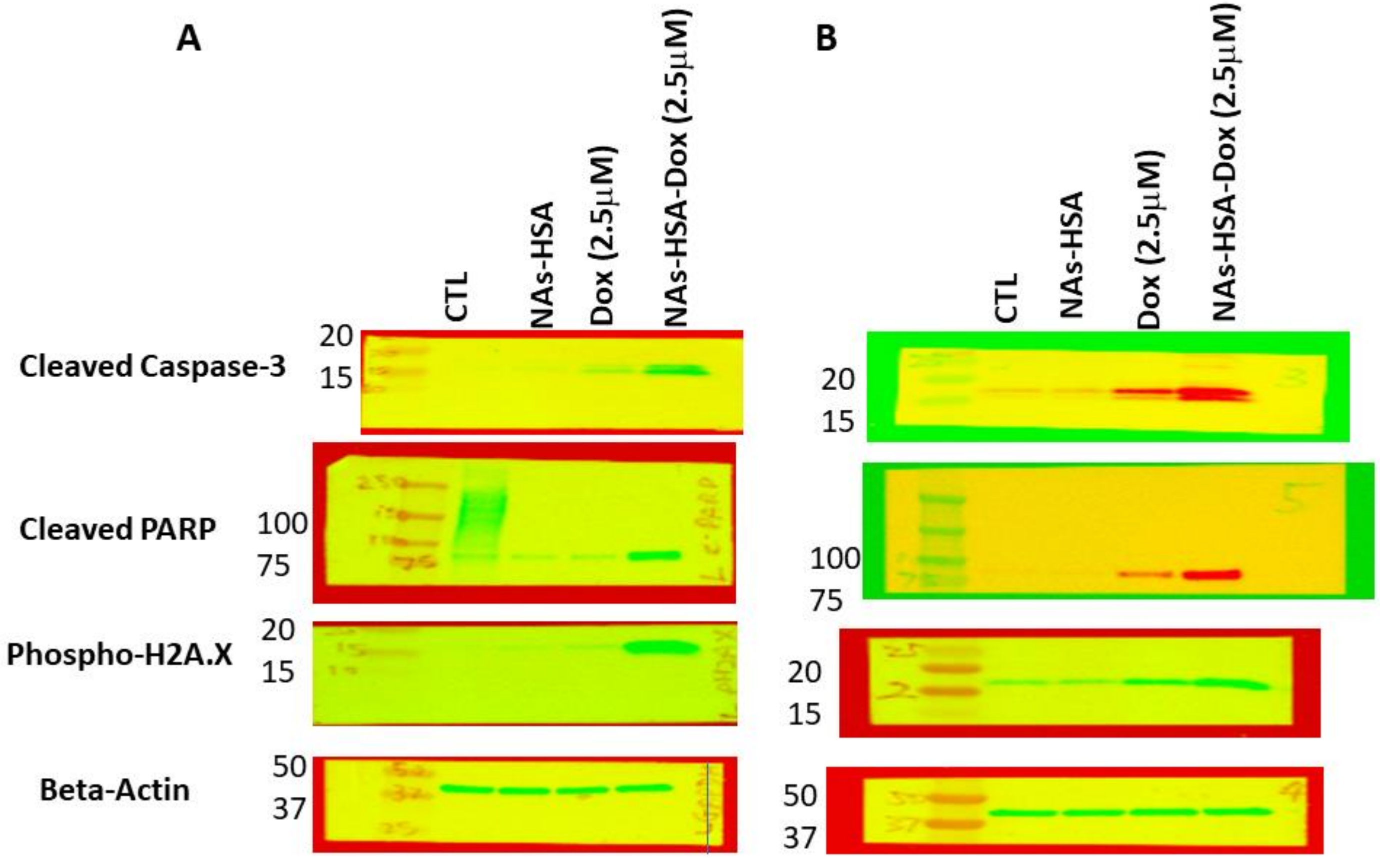

Figure S2: Original western blots for Figure 4.

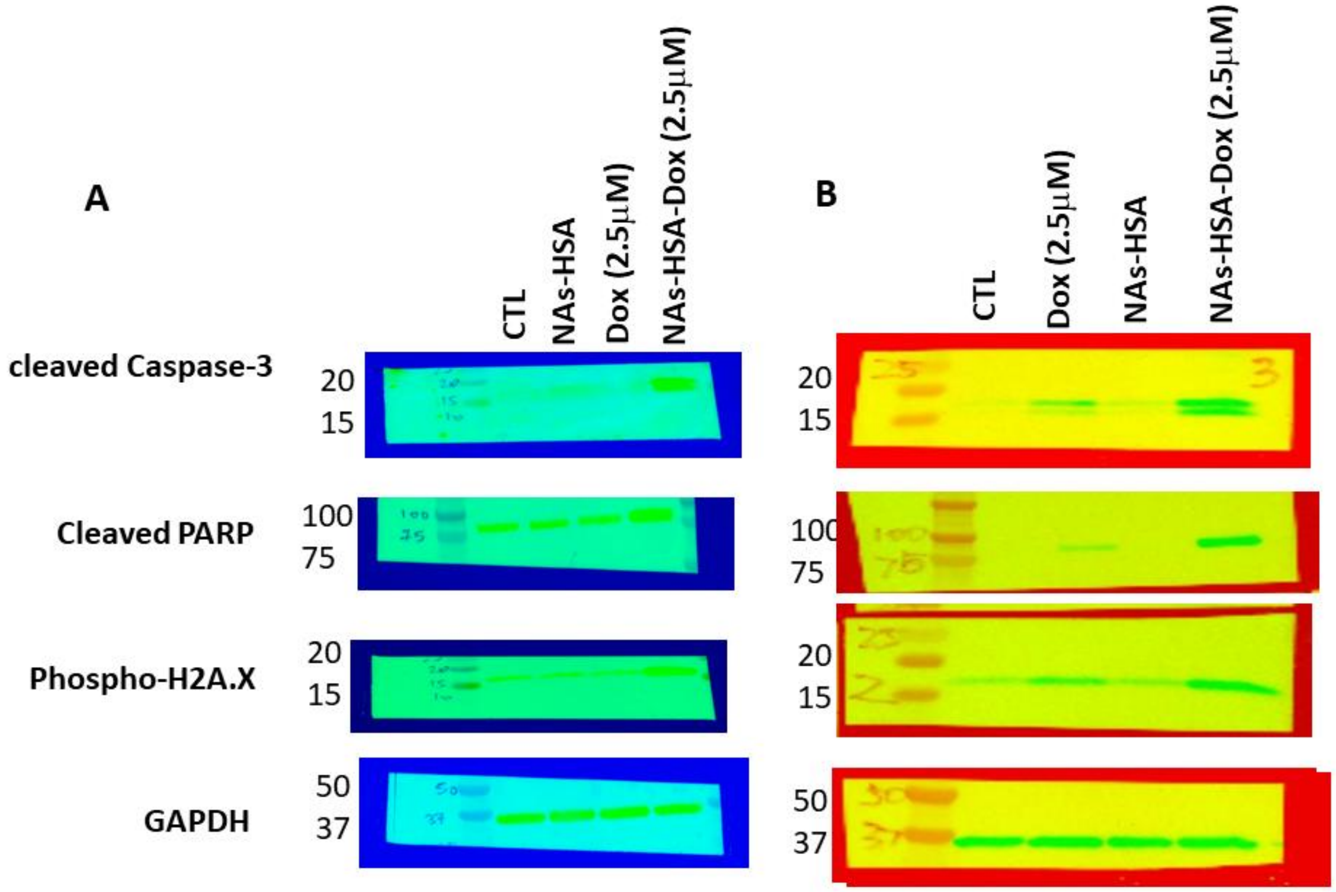

Figure S3: Original western blots for Figure S1.

Table S1. Kinetic parameters for DOX release obtained by applying the four kinetic models.

Sample	pH	Zero order (Eq. 3)		First order (Eq. 4)		Ritger-Peppas (Eq. 5)			Peppas-Sahlin (Eq. 6)			
		k	R^2	k	R^2	n	k	R^2	k_1	k_2 (10^2)	K_1/K_2	R^2
Nas-HSA	7.4	0.2227	0.5785	0.0086	0.9114	0.3010	0.0716	0.9749	0.0318	0.04	79.5	0.9823
	5.0	0.5107	0.4664	0.0118	0.9194	0.4495	0.0908	0.9433	0.0741	0.14	52.9	0.9790
Nas	7.4	0.5296	0.1760	0.0335	0.9849	0.0854	0.4410	0.7699	0.0716	0.14	51.1	0.7715
	5.0	0.6933	0.1464	0.0444	0.9922	0.0656	0.6399	0.8696	0.0915	0.18	50.8	0.6548
Dox	7.4	0.5441	0.1946	0.0337	0.9866	0.0895	0.4464	0.8010	0.0731	0.15	48.7	0.7749
	5.0	0.7213	0.1117	0.0457	0.9853	0.0587	0.6794	0.7155	0.0955	0.20	47.8	0.6622
